# Supplementary figures and images for: Peripheral blood methylation profiling of female Crohn’s disease patients
Source: Clin Epigenetics. 2016 Jun 8;8:65. doi: 10.1186/s13148-016-0230-5 (PMC4897922; doi:10.1186/s13148-016-0230-5)

a)

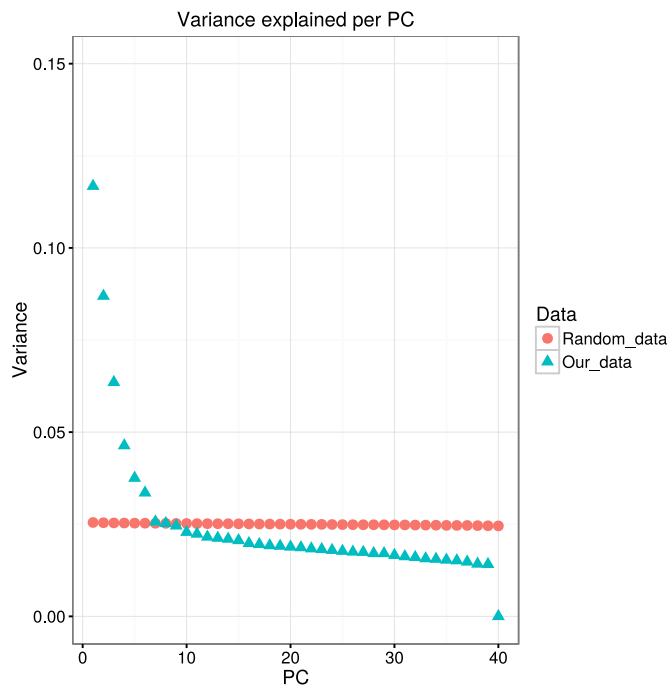

b)

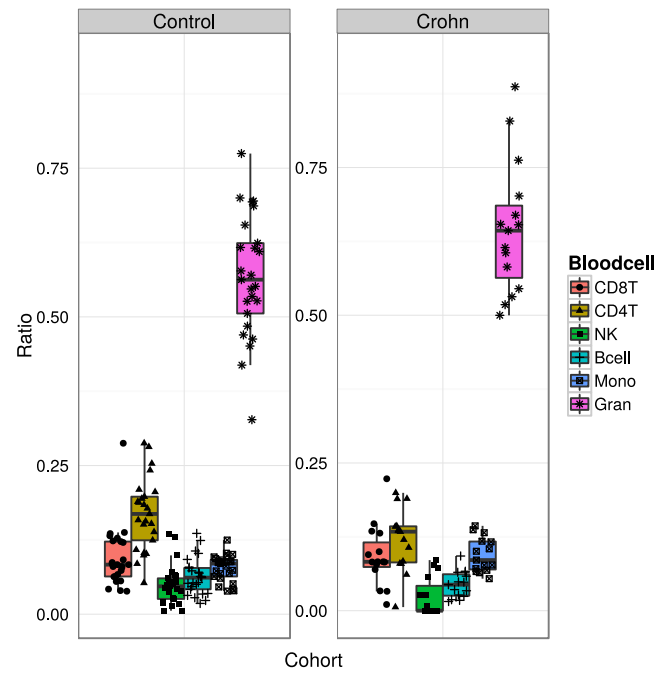

c)

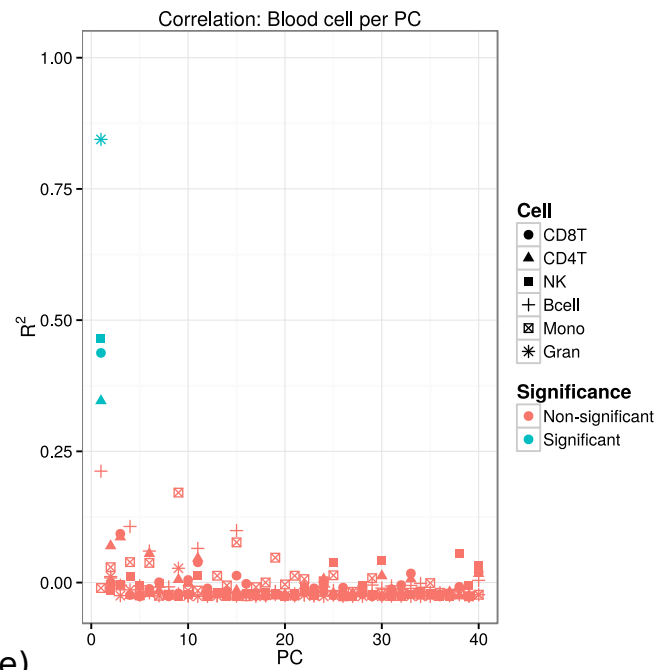

d)

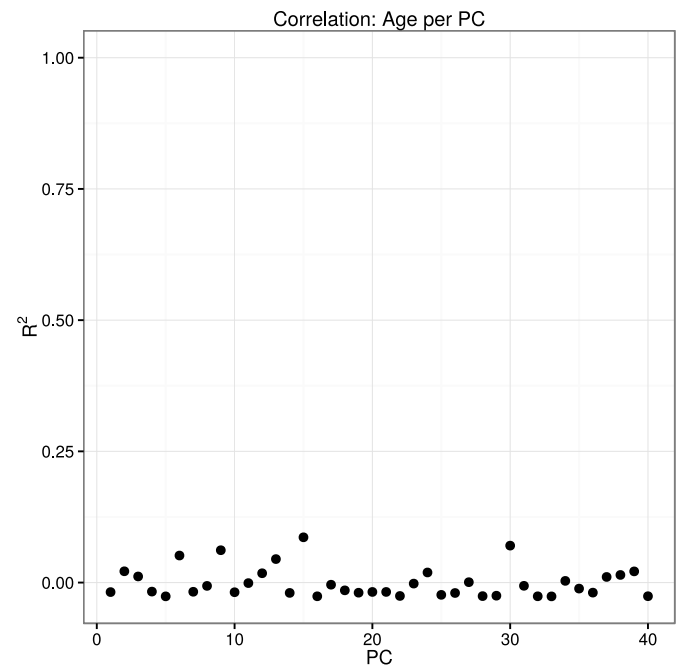

e)

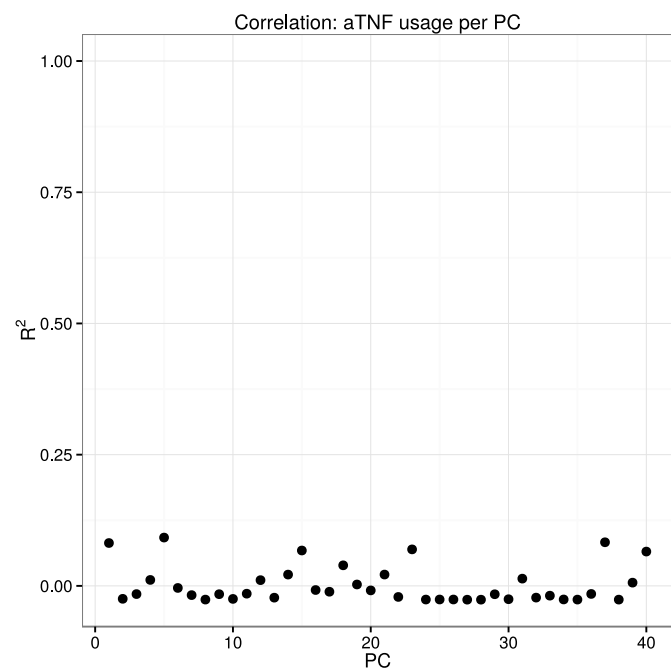

Supplement: Additional file 1: Figure S1. — Exploratory data analysis of putative biological confounders. a) Variance explained per principal component based on our 450k data (turquoise triangles) versus randomly generated data (red circles). b) Dot-boxplot of the cellular composition as estimated by the Houseman algorithm [22, 23]. c) Pearson correlation coefficient (R2) of each blood cell proportion with the principal components. The statistically significant correlations are indicated in turquoise, whereas statistically non-significant associations are indicated in red. Similar correlations were calculated for d) age and e) anti-TNF usage. (PDF 119 kb) [file 13148_2016_230_MOESM1_ESM.pdf]

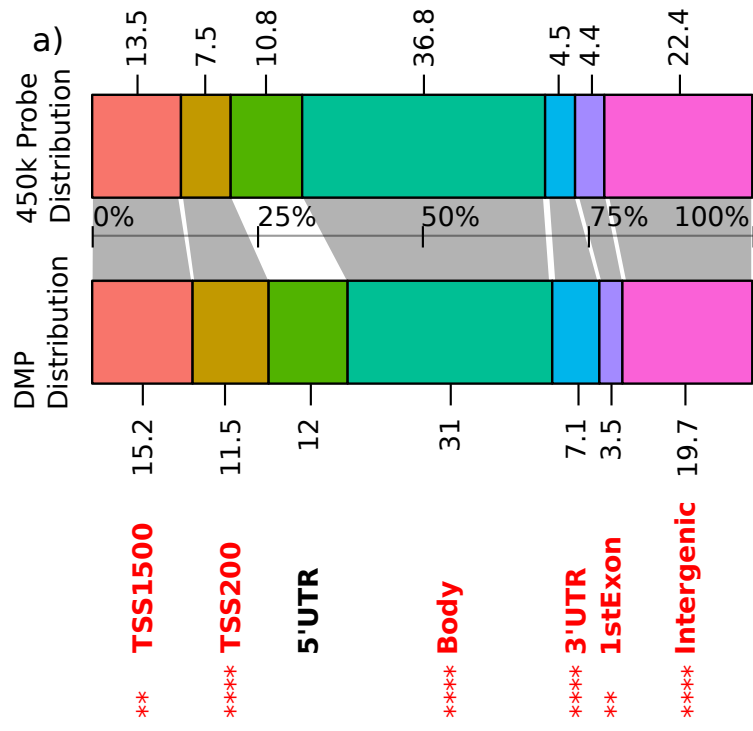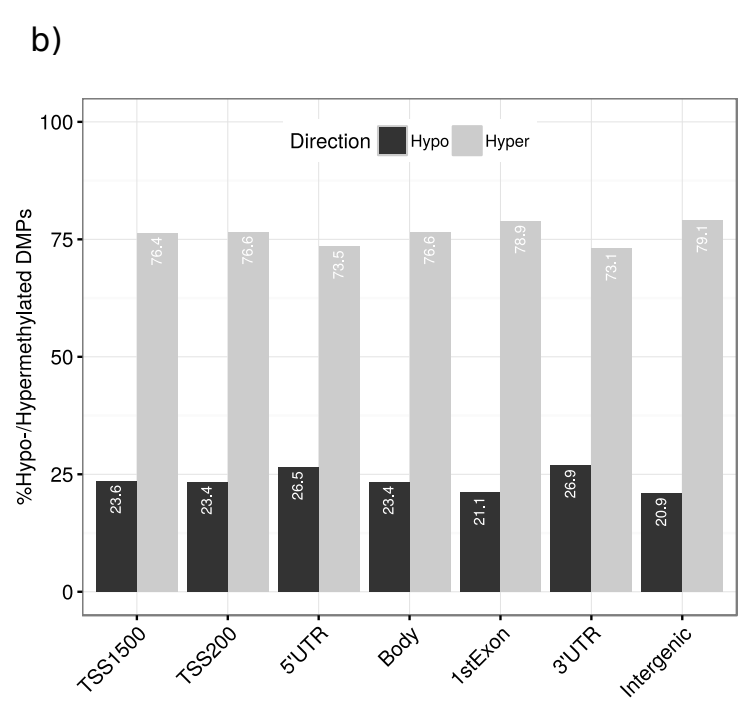

Supplement: Additional file 3: Figure S2. — DMP-distribution per genetic feature and per chromosome. a) Comparison of the probe distribution on the 450k versus the DMP distribution per genetic feature where the different colors represent the different genetic features. The numbers along the barplot represent the percentages of the 450k probes (top) or DMPs (bottom) per genetic feature. Significantly different DMP-distributions are indicated in bold red with the asterisks indicating the level of significance as found in Additional file 5: Table S3 (*: p < 0.05, **: p < 0.01, ***: p < 0.001, ****:p < 0.0001). b) For each genetic feature the percentage hypo- and hypermethylated DMPs is indicated with barplots in black and gray respectively. (PDF 54 kb) [file 13148_2016_230_MOESM3_ESM.pdf]

a)

HLA-J

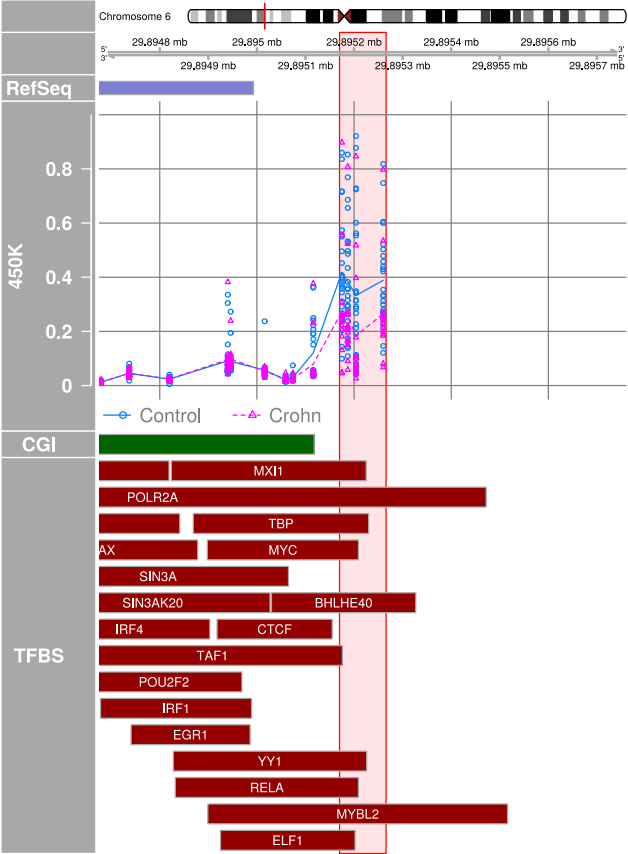

b)

MOV10L1

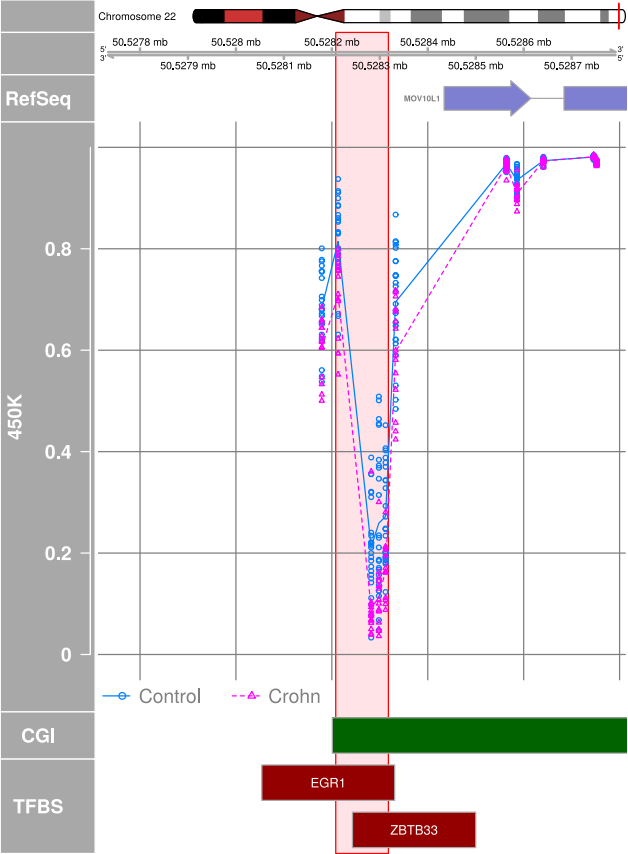

c)

LINC00612

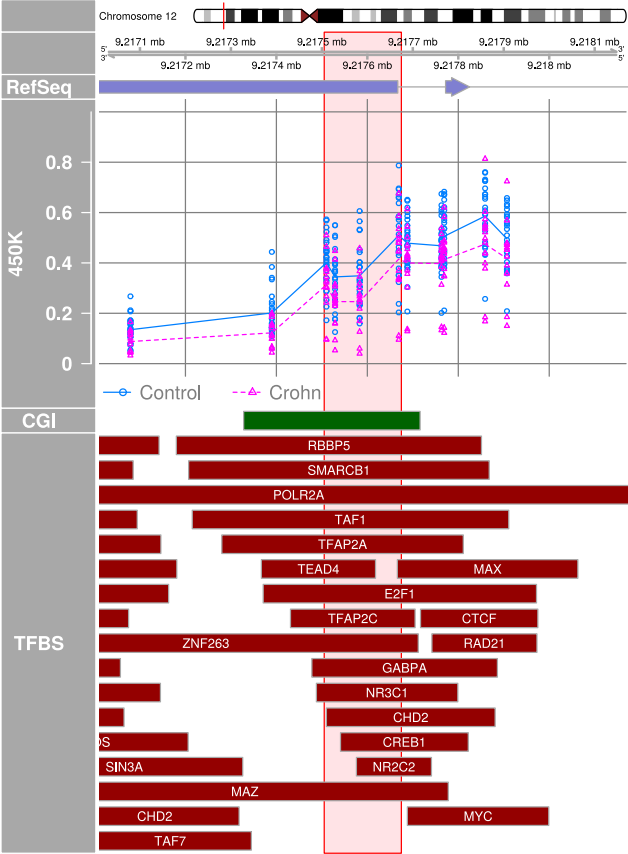

d)

SHANK2

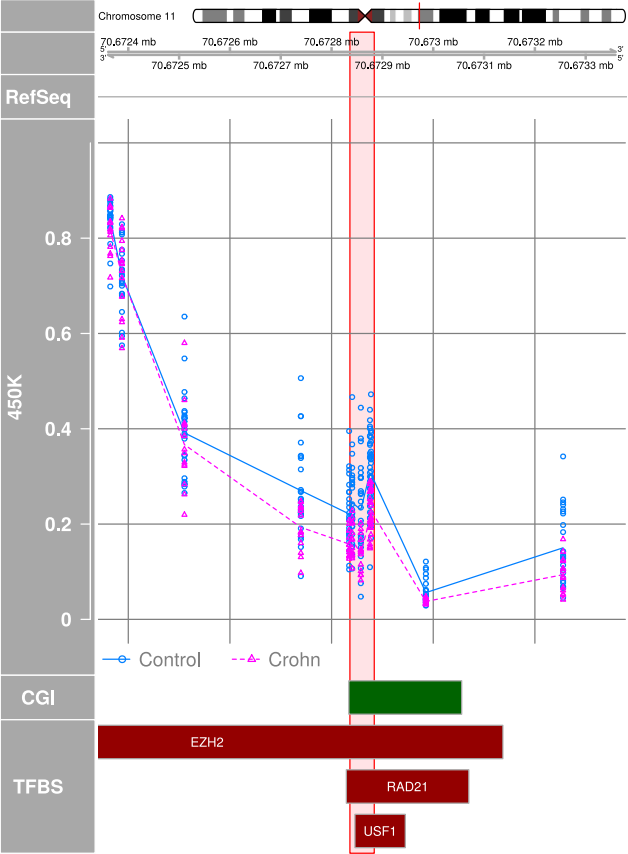

e)

APOBEC1

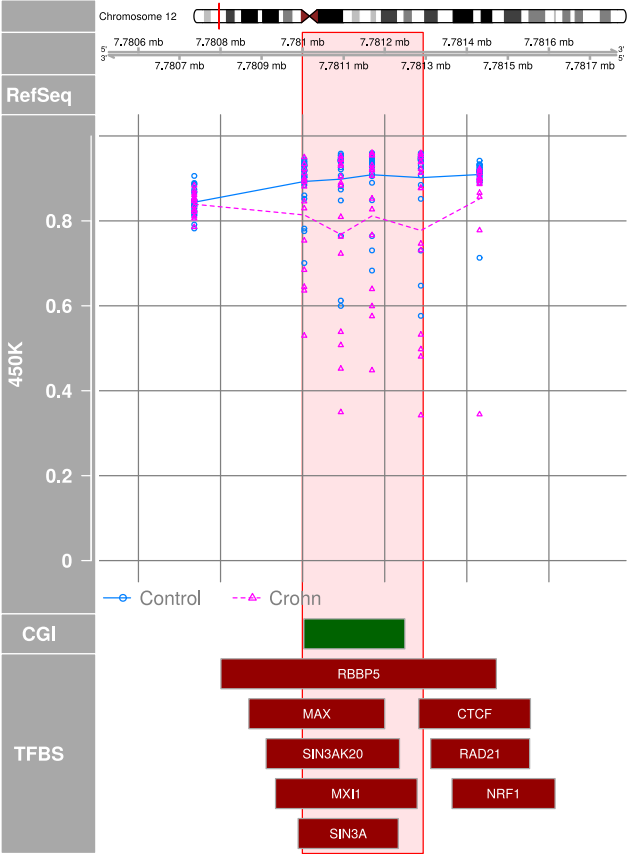

f)

BOLA3

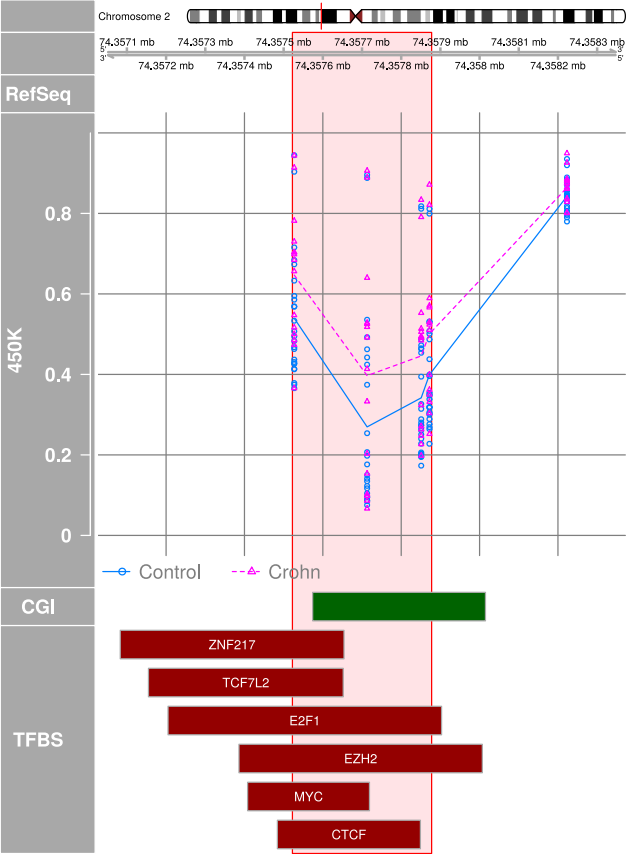

g)

OR2L13

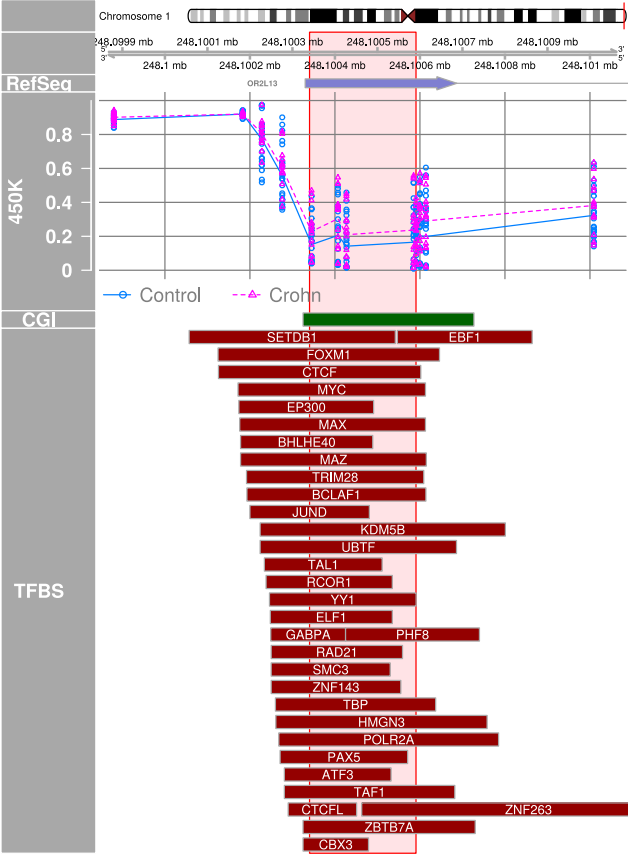

h)

TACSTD2

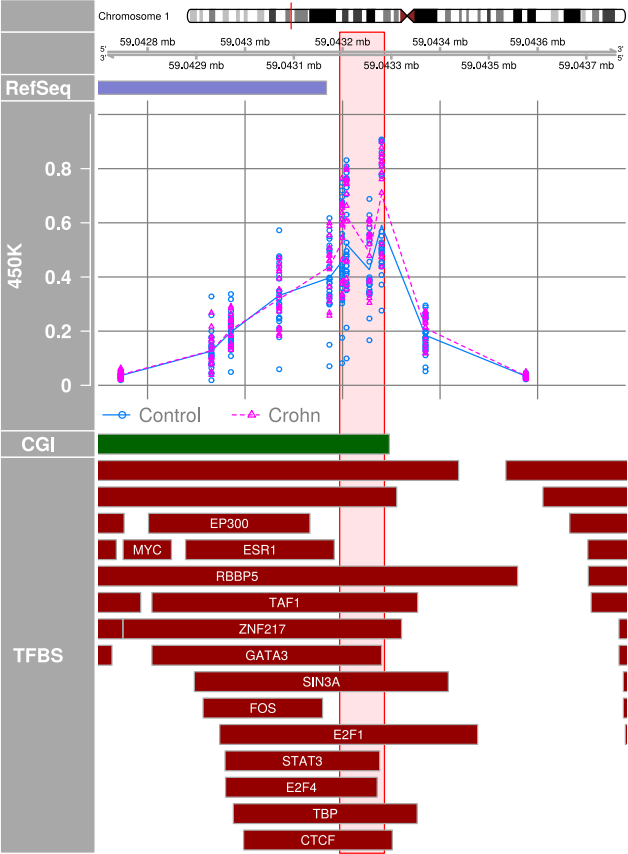

Supplement: Additional file 6: Figure S3. — Differentially methylated regions. Plots of the methylation levels of the DMRs nearest to: a) HLA-J, b) MOV10L1, c) LINC00612, c) SHANK2, d) APOBEC1, e) OR2L13 and f) TACSTD2 from the 450k (“450K”) superposed onto the RefSeq gene (“RefSeq gene”), the CpG island (“CGI”) and the transcription factor binding sites (“TFBS”), as retrieved from the UCSC Genome Browser. The red transparent rectangle indicates the DMR as reported by bumphunter. (PDF 420 kb) [file 13148_2016_230_MOESM6_ESM.pdf]

a)

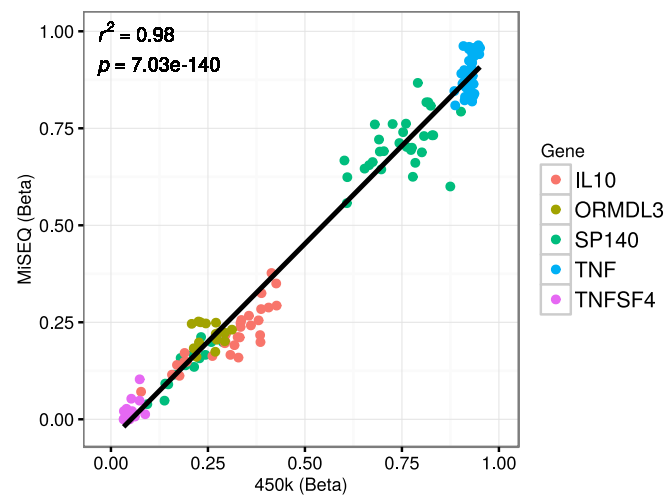

b)

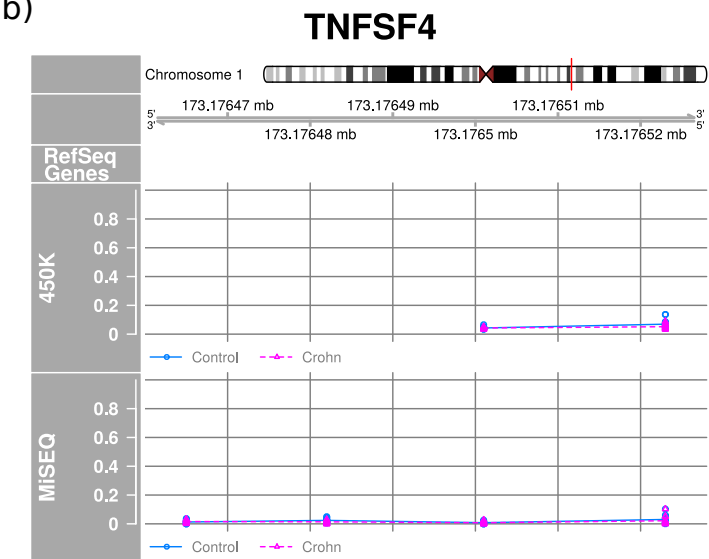

c)

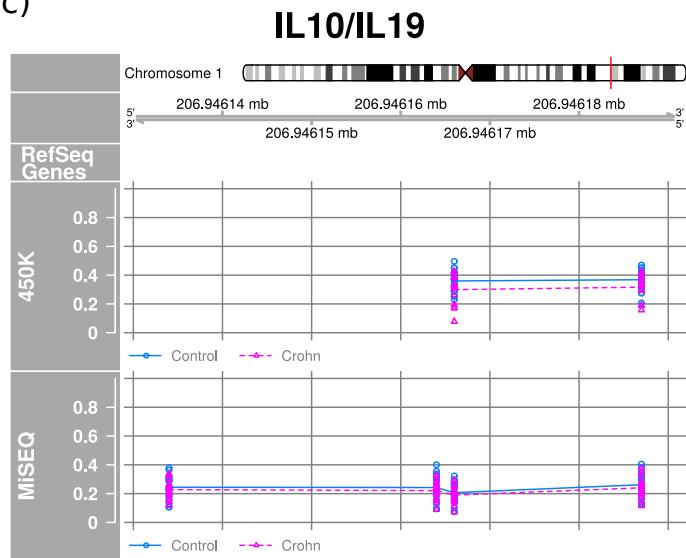

d)

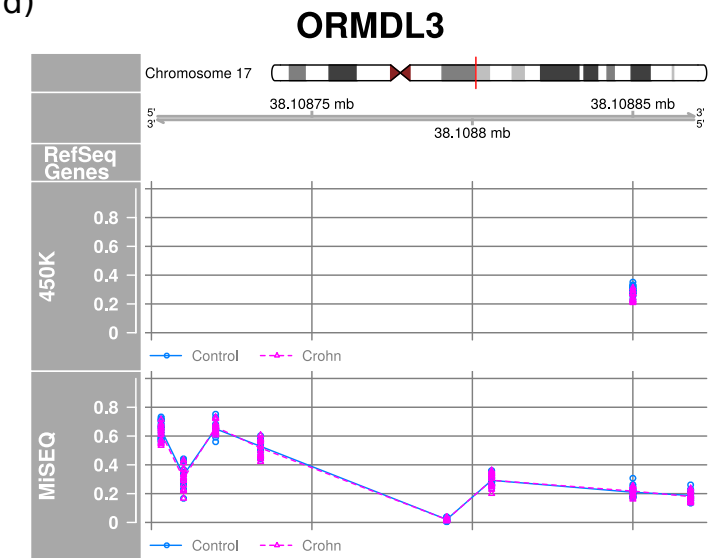

Supplement: Additional file 8: Figure S4. — MiSeq validation. a) Correlation of the methylation levels obtained from 450k and MiSeq. Each color represents the gene associated to the plotted CpG. Visualization of the methylation levels in beta of the DMPs obtained from the 450k (“450K”) compared to the methylation levels obtained from MiSeq sequencing (“MiSEQ”) superposed onto the RefSeq gene (“RefSeq gene”) for b) TNFSF4, c) IL10/IL19, and d) ORMDL3. (PDF 146 kb) [file 13148_2016_230_MOESM8_ESM.pdf]
