# Supplementary material for: Peripheral blood methylation profiling of female Crohn’s disease patients
Source: Clin Epigenetics. 2016 Jun 8;8:65. doi: 10.1186/s13148-016-0230-5 (PMC4897922; doi:10.1186/s13148-016-0230-5)
Supplement: Additional file 4: Table S2. — DMP-distribution statistics per genetic feature. Results are ordered by the first Fisher test (left three columns), which tests for differences in DMP-distribution per genetic feature, and the second Fisher test (right three columns), which tests for differences in the distribution of the hypo-/hypermethylated DMPs. Statistics provided are the odds ratios with the 95 % confidence intervals (“OR (CI-95)”), the p values and the Bonferroni-adjusted p values. (DOCX 53 kb) [file 13148_2016_230_MOESM4_ESM.docx]

|  | **DMP-distribution** | | | **Hypo-/hypermethylated DMP-distribution** | | |
| --- | --- | --- | --- | --- | --- | --- |
|  | **OR (CI-95)** | **pvalue** | **padj** | **OR (CI-95)** | **pvalue** | **padj** |
| **1stExon** | 0.62 (0.54-0.71) | 7.56E-14 | 5.29E-13 | 0.87 (0.64-1.17) | 0.39 | 1 |
| **3'UTR** | 1.29 (1.12-1.48) | 5.31E-04 | 3.72E-03 | 1.21 (0.94-1.56) | 0.13 | 0.91 |
| **5'UTR** | 0.89 (0.81-0.98) | 0.0144 | 0.10 | 1.20 (1.01-1.42) | 0.03 | 0.23 |
| **Body** | 1.30 (1.22-1.38) | 5.63E-18 | 3.94E-17 | 0.99 (0.89-1.11) | 0.87 | 1 |
| **Intergenic** | 1.18 (1.10-1.26) | 2.14E-06 | 1.50E-05 | 0.84 (0.71-0.98) | 0.03 | 0.21 |
| **TSS1500** | 0.87 (0.80-0.94) | 5.46E-04 | 3.82E-03 | 1.01 (0.85-1.19) | 0.90 | 1 |
| **TSS200** | 0.62 (0.56-0.69 | 2.81E-20 | 1.97E-19 | 1 (0.80-1.23) | 1 | 1 |
