# Supplementary material for: Peripheral blood methylation profiling of female Crohn’s disease patients
Source: Clin Epigenetics. 2016 Jun 8;8:65. doi: 10.1186/s13148-016-0230-5 (PMC4897922; doi:10.1186/s13148-016-0230-5)
Supplement: Additional file 5: Table S3. — DMP-distribution statistics per chromosome. Results are ordered by the first Fisher test (left three columns), which tests for differences in DMP-distribution per chromosome, and the second Fisher test (right three columns), which tests for differences in the distribution of the hypo-/hypermethylated DMPs. Statistics provided are the odds ratios with the 95 % confidence intervals (“OR (CI-95)”), the p values and the Bonferroni-adjusted p values. (DOCX 98 kb) [file 13148_2016_230_MOESM5_ESM.docx]

| **Chromosomes** | **DMP-distribution** | | | **Hypo-/hypermethylated DMP-distribution** | | |
| --- | --- | --- | --- | --- | --- | --- |
|  | **OR (CI-95)** | **pvalue** | **padj** | **OR (CI-95)** | **pvalue** | **padj** |
| **chr1** | 1.27 (1.16-1.39) | 7.51E-07 | 1.73E-05 | 0.77 (0.60-0.98) | 0.03 | 0.65 |
| **chr2** | 1.10 (0.98-1.23) | 0.10 | 1 | 1.07 (0.81-1.40) | 0.63 | 1 |
| **chr3** | 1.18 (1.03-1.34) | 0.01 | 0.28 | 1.05 (0.76-1.42) | 0.76 | 1 |
| **chr4** | 0.82 (0.69-0.97) | 0.02 | 0.38 | 0.95 (0.61-1.43) | 0.84 | 1 |
| **chr5** | 0.87 (0.74-1.00) | 0.06 | 1 | 0.76 (0.50-1.12) | 0.17 | 1 |
| **chr6** | 0.98 (0.87-1.10) | 0.70 | 1 | 0.90 (0.66-1.20) | 0.52 | 1 |
| **chr7** | 0.93 (0.81-1.06) | 0.27 | 1 | 1.12 (0.81-1.53) | 0.47 | 1 |
| **chr8** | 1.03 (0.89-1.20) | 0.65 | 1 | 0.91 (0.62-1.31) | 0.65 | 1 |
| **chr9** | 0.96 (0.76-1.19) | 0.78 | 1 | 0.77 (0.41-1.37) | 0.42 | 1 |
| **chr10** | 1.13 (0.99-1.29) | 0.07 | 1 | 0.92 (0.65-1.27) | 0.63 | 1 |
| **chr11** | 1.14 (1.01-1.29) | 0.03 | 0.71 | 1.38 (1.05-1.82) | 0.02 | 0.45 |
| **chr12** | 0.96 (0.83-1.10) | 0.57 | 1.00 | 1.39 (1.00-1.92) | 0.04 | 0.91 |
| **chr13** | 0.99 (0.81-1.20) | 0.96 | 1 | 0.81 (0.47-1.34) | 0.48 | 1 |
| **chr14** | 1.12 (0.94-1.32) | 0.18 | 1 | 0.82 (0.52-1.25) | 0.37 | 1 |
| **chr15** | 1.17 (0.99-1.38) | 0.05 | 1 | 1.11 (0.74-1.63) | 0.62 | 1 |
| **chr16** | 0.89 (0.75-1.04) | 0.13 | 1 | 1.26 (0.87-1.81) | 0.22 | 1 |
| **chr17** | 1.06 (0.93-1.21) | 0.34 | 1 | 0.82 (0.59-1.14) | 0.25 | 1 |
| **chr18** | 0.90 (0.66-1.20) | 0.53 | 1 | 1.61 (0.81-3.06) | 0.16 | 1 |
| **chr19** | 0.70 (0.59-0.82) | 3.18E-06 | 7.30E-05 | 0.98 (0.64-1.45) | 1 | 1 |
| **chr20** | 0.93 (0.74-1.15) | 0.56 | 1 | 0.73 (0.39-1.29) | 0.30 | 1 |
| **chr21** | 1.25 (0.91-1.67) | 0.13 | 1 | 1.39 (0.67-2.73) | 0.37 | 1 |
| **chr22** | 0.98 (0.76-1.23) | 0.91 | 1 | 0.50 (0.22-1.01) | 0.05 | 1 |
| **chrX** | 0.32 (0.22-0.46) | 1.33E-14 | 3.06E-13 | 9.18 (4.08-22.62) | 2.42E-09 | 5.56E-08 |
