# Supplementary material for: Peripheral blood methylation profiling of female Crohn’s disease patients
Source: Clin Epigenetics. 2016 Jun 8;8:65. doi: 10.1186/s13148-016-0230-5 (PMC4897922; doi:10.1186/s13148-016-0230-5)
Supplement: Additional file 9: Table S6. — Comparison of the top DMPs reported by McDermott et al. with our own data. The location data: Illumina probe ID, chromosome, position, and associated gene are shown alongside the effect size per study. CpGs where opposite effects were found are indicated in bold. (DOCX 15 kb) [file 13148_2016_230_MOESM9_ESM.docx]

| **Probe ID** | **DMP location (hg19)** | **Associated gene** | **Effect size** | | |
| --- | --- | --- | --- | --- | --- |
|  |  |  | **McDermott et al.** | | **Our data** |
|  |  |  | **CD** | **UC^1^** | **(CD)** |
| cg16176675 | chr5:134786942 | TIFAB | 0.092 | 0.084 | 0.201 |
| cg21442182 | chr13:31446420 |  | 0.092 |  | 0.260 |
| cg10311161 | chr8:68110902 | ARFGEF1 | 0.102 | 0.086 | 0.190 |
| cg17579089 | chr3:47269695 | KIF9 | 0.09 |  | 0.185 |
| cg05025332 | chr2:65165160 |  | 0.103 |  | 0.154 |
| cg20061654 | chr22:45608492 | C22orf9 | 0.081 | 0.074 | 0.168 |
| cg21935981 | chr22:45608465 | C22orf9 | 0.075 | 0.068 | 0.060 |
| cg22013370 | chr1:241981543 |  | 0.105 |  | 0.167 |
| cg05921138 | chr2:121440849 | SORL1 | 0.102 |  | 0.128 |
| **cg02619001** | **chr17:47592333** | **NGFR** |  | **0.075** | **-0.011** |
| **cg07795766** | **chr22:45608516** | **C22orf9** |  | **0.051** | **-0.060** |
| **cg23320499** | **chr12:12659177** | **DUSP16** |  | **-0.077** | **0.113** |
| cg04223044 | chr22:45608428 | C22orf9 |  | 0.072 | 0.025 |
| cg19380303 | chr15:90172193 | KIF7 |  | 0.082 | 0.144 |
| cg04407417 | chr12:52228651 |  |  | 0.041 | 0.150 |
