# Supplementary material for: Peripheral blood methylation profiling of female Crohn’s disease patients
Source: Clin Epigenetics. 2016 Jun 8;8:65. doi: 10.1186/s13148-016-0230-5 (PMC4897922; doi:10.1186/s13148-016-0230-5)
Supplement: Additional file 10: Table S7. — MiSeq primers. The primer data used for MiSeq amplicon sequencing is shown alongside the included 450k probes. (DOCX 16 kb) [file 13148_2016_230_MOESM10_ESM.docx]

| **Primer name** |  | **Sequence (5’ to 3’)** | **Included 450k probes** |
| --- | --- | --- | --- |
| SP140_P1 | F | TGGTAGGAATAAGTGATTTTGTTTTT | cg17607231, cg12976883 and cg03887528 |
|  | R | CTCCACTTACCATCTACCCCTACTA |  |
| SP140_P2 | F | TAGGGGTAGATGGTAAGTGGAGATA | cg05564251 and cg04579254 |
|  | R | CCAATCCAACCATTTACAAATATAAA |  |
| TNF_P3 | F | GGAGTTGTTTTGGTTTAGATATGTTTT | cg01360627, cg23384708, cg20477259 |
|  | R | AAAAACTTAAAAAACTTCTTCCCAC |  |
| TNFSF4 | F | ATTTTTTGTTAGTTTAGAGGAAAAA | cg02137984, cg06825478, cg17755321 |
|  | R | AAAAATTAAACCCTTTCCATCTTC |  |
| ORMDL3_P2 | F | GATTGAATTGTGGGTTTAGGATAGT | cg12655416 |
|  | R | TATATCCCTTTCCCCTAAACAAAAT |  |
| IL10_19_P1 | F | TGGTAGGAATAAGTGATTTTGTTTTT | cg02965290 |
|  | R | CTCCACTTACCATCTACCCCTACTA |  |

.
